# Against the Pendulum Movement, in Working the Midwifery Forceps

**Published:** 1876-03

**Authors:** J. Matthews Duncan


### MISCELLANEOUS.

### Against the Pendulum Movement in Working the Midwifery Forceps.

By J. MATTHEWS DUNCAN, M. D.

My object in writing this short paper is to contribute to the conclusive giving up of a bad practice. I have long publicly taught that the pendulum movement is useless and injurious; but that kind of publicity is not so wide as desirable. Others, of great authority, from their recognised science and experience, such as Litzmann of Kiel, have made objection to this manœuvre, but it is still extensively used and vigorously recommended. There is, indeed, a natural tendency to resort to this swinging motion in the hands or minds of many practitioners, who probably have erroneous notions of what is called gomphosis, and think of drawing a nail out of a board. Besides, the movement is an element in that fussiness which recommends itself to many, if not the majority, of practitioners, and satisfies the admiring onlookers. The solemn arguments by which obstretric authors support its use I shall presently discuss.

My objection does not extend to changes in the direction of traction by the forceps, such as may be required according as the dragged head descends, or such as may be called for when the head has been previously inefficiently dragged in a wrong direction. It is to the changes implied in the oscillatory or leverage or pendulum-like movement of the dragging instrument that my objections are raised. These movements I regard as not merely useless, but as also injurious. They have been considered as lending to economy of force; but this is the opposite to the truth, as I shall hereinafter show. I mention this imagined advantage now in order to say that, were it true only so far as the practitioner is concerned, I would esteem such economy as of no value, for the accoucheur is present with a practically boundless supply of force, which he is willing to lavish for behoof of his patient.

In the following remarks I shall make reference only to that pendulum movement from side to side, which alone is, so far as I know recommended in these latter times. The pendulum movement in a sagittal direction, as recommended by the early describers of the forceps operation, is still more open to objections than the former.

In describing or defending the pendulum movement, two great points are made: first, that it is analogous to, or idential with, that of a lever and double rack; and, secondly, that by resorting to it there in an economy of force.

The lever and double rack hypothesis may be considered first. It is difficult to dispose of it only because it is so flimsy that it is impossible to get hold of it. There is no toothed rack on the wall of the pelvis. There is no roughness to take the place of a toothed rack. Were there any such teeth or roughness, the worst use that could be made of them would be to make them assume the function of a rack. The accoucheur should strive to advance the head as smoothly and with as little impression as possible on the walls of the pelvis. Further, there are no teeth or roughness on the fetal head to fit into the teeth of the supposed rack.

Without the lever and double rack hypothesis substantiated, the movement must be useless; for it is, and must be, done to no purpose. It is conceivable that the head may be sized with such a degree of firmness by the blades of the forceps as to be moved or made to revolve as on a pivot; but such movement, without the additional force required in simple and successful traction, uncomplicated by the movement, would be of no avail. It would not advance the head. Without the lever and double rack, it would only cause revolutions as on a pivot—it would not produce progress.

Let us imagine that the accoucheur, dragging to one side, produces advance of the opposite side, which he maintains by continued dragging, while he makes the oscillatory movement in order to drag to the other side. In this way he may make the head advance while using an oscillatory movement. Every one knows that this is easily done. But then every one must also recognise the utter inutility of the movement. It is the imitation of the action of a lever and double rack without a trace of its utility. An unnecessary movement like this has great disadvantages. The pressure exerted, and the traction force used, are probably greater, certainly not less, than if simple traction were exerted to produce the desired result. Pulling the head down at one side and then at the other, and so advancing, is merely an injuriously complicated way of producing simple progress. It produces no evasion nor diminution of the difficulty to be overcome, while it has concomitant and avoidable evils.

In answer to this reasoning one might refer to an analogy in the way in which a cork is sometimes extracted from a bottle without using a corkscrew. But a study of this analogy only confirms the argument. For the cork would be better, and on the whole easier, extracted by simple uncomplicated traction as by a corkscrew. Besides, the cork and the bottle mutually exert such friction force as prevents retraction of one side if advanced, just as if the bottle were a rack and the cork the lever; and such is in no sense the case with the fetal head and pelvis. Lastly, this oscillatory advance of the cork is only sought when the power fails to produce a direct advance, and, in the case of the forceps, there is never deficiency of power; for as we have already said, the power applicable by the accoucheur is practically boundless, or, in more sober terms, it is greater than he can safely apply.

The idea that there is any saving of force, so far as pressure on the mother's and child's parts are concerned, by resort to the oscillatory or pendulum movement, is such that I cannot argue against it. The question involved is purely mechanical and of extreme simplicity. It is this: a mechanical difficulty in bringing a child's head through a resisting passage has to be overcome; further, the difficulty is not to be evaded by changing the position of the child's head; on the contrary, that position may be supposed to be the most favourable for facility of propulsion. Now, can any oscillation or other imaginable movement diminish the mutual, and, in this case, injurious pressure or force required to produce advance? The question requires no answer. The supposition is absurd. Moreover, the absurdity is not less, whatever phrase may be given to the hypothetical advantages of the pendulum movement. A certain amount of work has to be done; the heaed has to be advanced against resistance that must be overpowered if the efiort is unsuccessful. Direct uncomplicated traction does the work in the simplest way, and no complication of it by pendulum movement or other can diminish the amount of work expended below that required by simple traction, The complication of simple traction may, however, increase the expenditure of force to a great extent.

The pendulum movement necessarily involves an injurious amount of pressure and consequent friction, in all cases, between the parts of the head to which the blades of the forceps are applied and the adjacent maternal structures. No doubt this friction is in most cases so slight and temporary as to be of little moment. But in some cases, when the resistance to progress arises from tight and undilatable soft parts, it may be very injurious. The most important forceps cases, however, are those where the obstacle to progress arises from hard parts; and, of these cases, the most frequent and characteristic are those of simple narrow or flat pelvis. In such cases the head has to be slowly dragged and perhaps moulded between the promontory of the sacrum and the pubic bones. Now here the pendulum movement involves special evils and dangers; for by it there is necessarily produced, besides the trivial friction, which is most extensive at the points where the blades are applied, a violent and powerful squeezing of the soft parts between the head and the opposing pelvic bones, on which the head works. This combination of wriggling and squeezing is altogether unnecessary, and must greatly aggravate the necessary or unavoidable mutual pressure, which is bad enough.

If for the carrying out of the plan of pendulum movement the forceps is made to compress the head so strongly as not to slip on it, which mode is probably regarded as desirable, then the points of the forceps, and especially the point of that blade which is on the side of the head towards which the movement is given, will exert a specially powerful, and certainly undesirable, amount of pressure on the parts of the child's head or face which they touch. If, on the other hand, the blades do not press the head with such firmness as to obviate a to-and-fro motion of them on the head, then the scalp will be liable to be much injured, and its surface abraded; conditions which are often observed as the result of this kind of proceeding.

There is in the mechanism of delivery, whether natural or morbid, nothing analogous to this artificially proceeded oscillating or pendulum motion. Nature pushes a fetal head through an obstructing passage by force, which produces, or may produce, on the one hand, dilatation or laceration of the passage; and, on the other hand, various kinds of changes in the shape and size of the fetal head. Our best guid in forceps cases is the process of nature; and it is probable that any future improvements in the working of the instrument will be the consequence of closer and successful investigation of the mechanism of labour in these difficult cases. The pendulum movement, in working the forceps, does not advantageously increase the power of the instrument to produce desired results.

The use of the forceps is to contribute, by artificial pulling, to the strength of the natural expulsive efforts which push. To this traction, judiciously applied, the practitioner should confine himself. The oscillatory movement will contribute nothing to the forward traction, and it is forward traction which alone is desirable.

In corroboratioh of these theoretical views as to the injuriousness and inutility of the pendulum movement in the working of the midwifery forceps, I might appeal to the extensive experience of myself and of many other practitioners. But such appeal can only be held as evidence sufficient to show that the pendulum movement is not necessary. It affords no evidence that using it or abstaining from it is the preferable plan. And I cannot imagine any method available at present whereby the results of experience can be made suitable for the final settlement of the matter.

Both plans are used by good practitioners. Traction without oscilliation is simple, effective, and in accordance with the method of nature's own efforts. Traction with oscillation is complicated; and many theoretical and practical objections may be made to it.

Other objections might be abduced against the pendulum movement, and some are candidly stated by authors who recommend its adoption. I have confined myself to the discussion of its supposed utility and inevitable evils. The only advantage which I can conceive it to possess, is one to which no one will avow his indebtedness, for it would be an admission of culpable want of knowledge, and consequent unjustifiable practice. A practitioner ignorant as to the proper direction of pulling may, by this motion of the forceps while extracting, fall accidentally upon the right direction, and thus do some good by mere luck, and at much risk, which should have been done intelligently, and without avoidable risk. It is, perhaps, in order to insure this kind of possible success, that same authors recommend the movement to be not pendulum-like, but rotatory, or in the grand so-called tours."

*Obstet. Journ. Gt. Britain and Ireland.*

